# Detection of the Japanese encephalitis vector mosquito *Culex tritaeniorhynchus* in Australia using molecular diagnostics and morphology

**DOI:** 10.1186/s13071-021-04911-2

**Published:** 2021-08-18

**Authors:** Bryan D. Lessard, Nina Kurucz, Juanita Rodriguez, Jane Carter, Christopher M. Hardy

**Affiliations:** 1 Australian National Insect Collection, National Research Collections Australia–CSIRO, GPO Box 1700, Canberra, ACT 2601 Australia; 2 Medical Entomology, NT Health–Royal Darwin Hospital, Top End Health Service, GPO Box 41326, Casuarina, NT 0810 Australia; 3 CSIRO Land and Water, GPO Box 1700, Canberra, ACT 2601 Australia

**Keywords:** *Culex* Vishnui subgroup, DNA barcoding, Phylogenetics, Northern Territory, Taxonomy

## Abstract

**Background:**

*Culex* (*Culex*) *tritaeniorhynchus* is an important vector of Japanese encephalitis virus (JEV) affecting feral pigs, native mammals and humans. The mosquito species is widely distributed throughout Southeast Asia, Africa and Europe, and thought to be absent in Australia.

**Methods:**

In February and May, 2020 the Medical Entomology unit of the Northern Territory (NT) Top End Health Service collected *Cx. tritaeniorhynchus* female specimens (*n* = 19) from the Darwin and Katherine regions. Specimens were preliminarily identified morphologically as the Vishnui subgroup in subgenus *Culex*. Molecular identification was performed using cytochrome *c* oxidase subunit 1 (COI) barcoding, including sequence percentage identity using BLAST and tree-based identification using maximum likelihood analysis in the IQ-TREE software package. Once identified using COI, specimens were reanalysed for diagnostic morphological characters to inform a new taxonomic key to related species from the NT.

**Results:**

Sequence percentage analysis of COI revealed that specimens from the NT shared 99.7% nucleotide identity to a haplotype of *Cx. tritaeniorhynchus* from Dili, Timor-Leste. The phylogenetic analysis showed that the NT specimens formed a monophyletic clade with other *Cx. tritaeniorhynchus* from Southeast Asia and the Middle East. We provide COI barcodes for most NT species from the Vishnui subgroup to aid future identifications, including the first genetic sequences for *Culex* (*Culex*) *crinicauda* and the undescribed species *Culex* (*Culex*) sp. No. 32 of Marks. Useful diagnostic morphological characters were identified and are presented in a taxonomic key to adult females to separate *Cx. tritaeniorhynchus* from other members of the Vishnui subgroup from the NT.

**Conclusions:**

We report the detection of *Cx. tritaeniorhynchus* in Australia from the Darwin and Katherine regions of the NT. The vector is likely to be already established in northern Australia, given the wide geographical spread throughout the Top End of the NT. The establishment of *Cx. tritaeniorhynchus* in Australia is a concern to health officials as the species is an important vector of JEV and is now the sixth species from the subgenus *Culex* capable of vectoring JEV in Australia. We suggest that the species must now be continuously monitored during routine mosquito surveillance programmes to determine its current geographical spread and prevent the potential transmission of exotic JEV throughout Australia.

**Graphical abstract:**

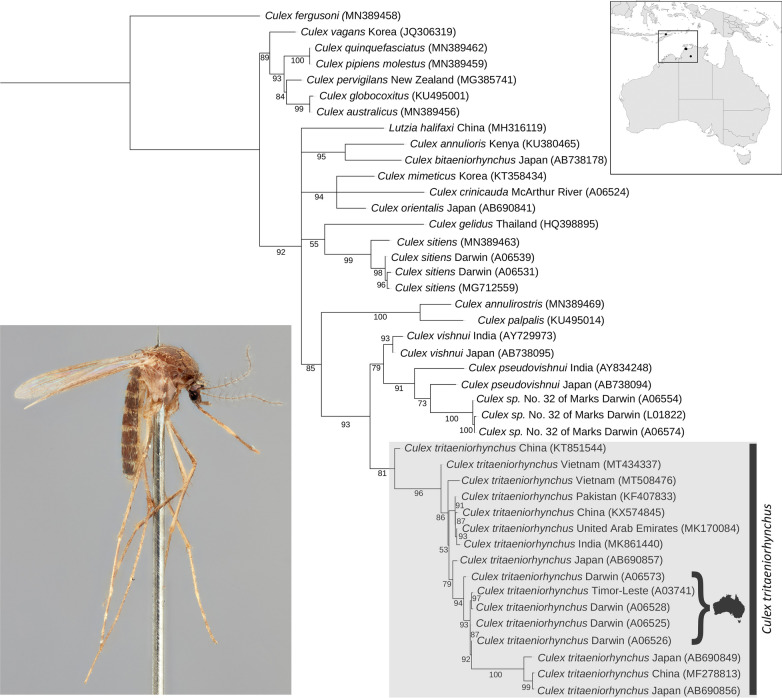

**Supplementary Information:**

The online version contains supplementary material available at 10.1186/s13071-021-04911-2.

## Background

*Culex* (*Culex*) *tritaeniorhynchus* Giles, 1901 is a widespread mosquito species occurring throughout Southeast (SE) Asia and extending into the Middle East, Africa and Europe [1], but is considered absent from Australia [2, 3]. The vector is a concern to health officials as the species is an important vector of Japanese Encephalitis virus (JEV; Flaviviridae: *Flavivirus*). This arbovirus is the leading cause of viral encephalitis in humans, with 68,000 cases reported globally each year, resulting in 20,400 deaths (25% mortality rate) and 14,000–24,000 neurological impairments, many of which occur in children under the age of 12 years [4, 5]. JEV also affects animals that act as reservoir hosts, including birds, cows, pigs, horses and other domestic animals [6–10], and can cause reproduction disorders and abortions in pigs [7].

*Culex tritaeniorhynchus* belongs to the subgenus *Culex* Linnaeus, 1758 and is a member of the Vishnui subgroup that comprises nine recognised species in Australasia: *Culex* (*Culex*) *pseudovishnui* Colless, 1957 and *Culex* (*Culex*) *vishnui* Theobald, 1901, both from SE Asia; *Culex* (*Culex*) *omani* Belkin, 1962 (Solomon Islands), *Culex* (*Culex*) *incognitus* Baisas, 1938 (Indonesia, Philippines), *Culex* (*Culex*) *perplexus* Leicester, 1908 (SE Asia, including Papua New Guinea); and the undescribed species *Culex* (*Culex*) No. 32, No. 68 and No. 92 of Marks from Australia [2, 11]. Three species in the Vishnui subgroup are confirmed JEV vectors: *Cx. pseudovishnui*, *Cx. tritaeniorhynchus* and *Cx. vishnui* [9]. Only two species from this subgroup are currently recognised as occurring in the Northern Territory (NT) of Australia: *Cx.* No. 32 and *Cx.* No. 92. The Australian species *Culex* (*Culex*) *crinicauda* Edwards, 1921, also occurring in the NT, was once considered as belonging to the Vishnui subgroup by Marks [11] but was later excluded from the group by subsequent taxonomists [2].

Accurate species identification of Australian mosquitoes is hindered by the lack of working taxonomists and presence of species complexes, cryptic species, rarely collected species, fragile specimens and a remarkable 170 undescribed species with unconfirmed vector status [2, 11–13]. Although more than 220 mosquito species are described from Australia [14], mosquito taxonomy did not significantly progress following the publication of the last volume of* The Culicidae of the Australasian Region* [15]. Regarding the Vishnui subgroup from the NT, *Cx. crinicauda*, *Cx.* No. 32 and *Cx.* No. 92 remain difficult to morphologically differentiate. Therefore, DNA barcoding of the cytochrome c oxidase subunit 1 (COI) gene is often routinely used to identify troublesome species to species level [9, 16].

Here, we report the first confirmed records of *Cx. tritaeniorhynchus* from Australia using DNA barcoding and morphology, using specimens collected from the NT. We provide DNA barcodes, high-resolution images and a taxonomic key to the adult females of Vishnui subgroup from the NT to improve species identification for future monitoring of potential JEV vectors in Australia.

## Methods

### Specimen collection

**Fig. 1:**
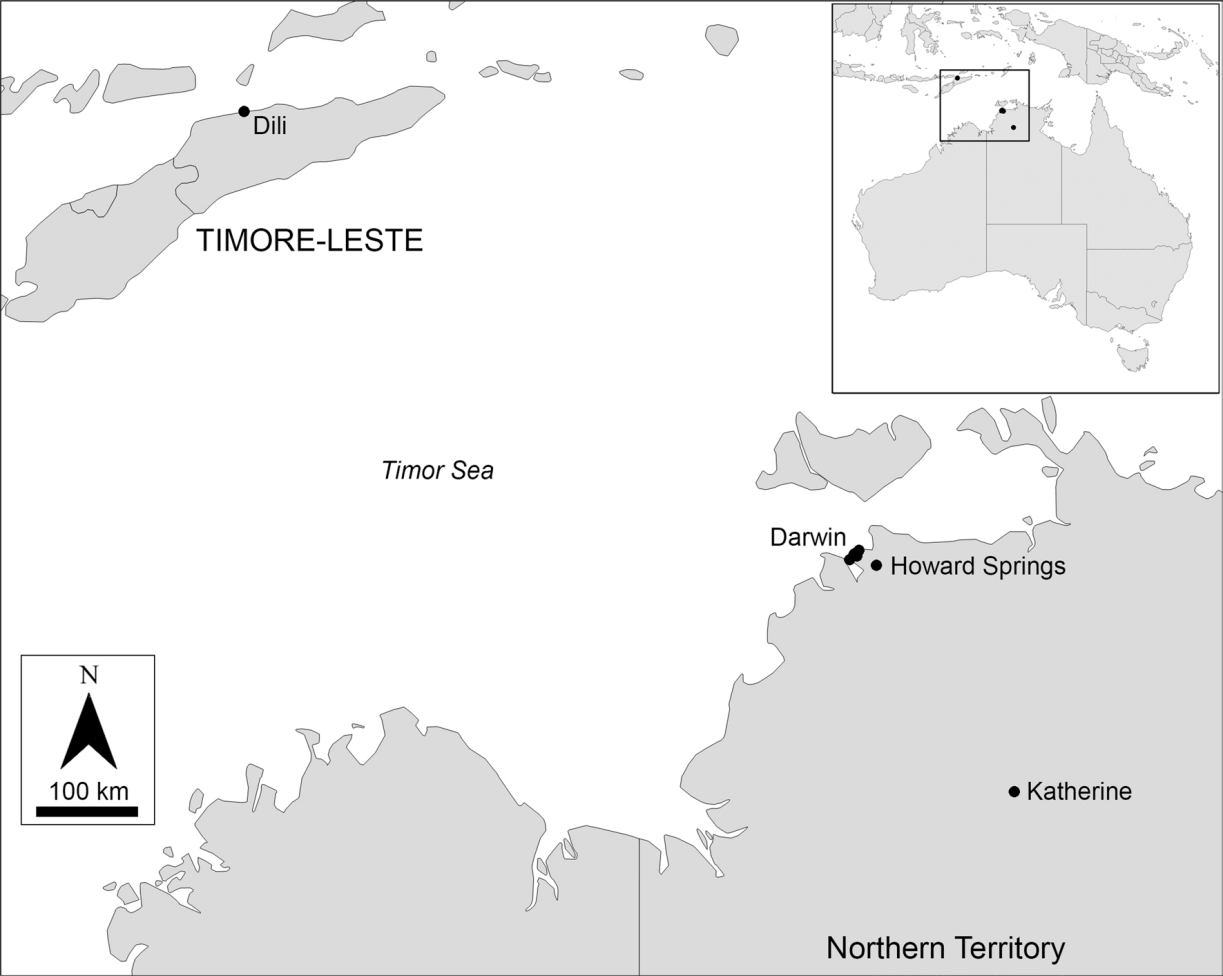
Distribution of *Culex* (*Culex*) *tritaeniorhynchus* specimens sequenced in this study

Between February and May 2020, 33 adult female mosquitoes were collected from the Darwin and Katherine regions in the NT, as part of the Medical Entomology (ME) Top End Health Service NT mosquito surveillance programme (Fig. 1; Additional file 1: Table S1); these were preliminarily identified as belonging to the Vishnui subgroup using the key to females of the subgenus *Culex* from the Australasian Region provided by Lee et al. [2]. Additional specimens included one larva collected using a 250-ml dipper and preserved in 100% ethanol from Howard Springs (Darwin region), identified as *Cx.* sp. No. 32 of Marks, and an adult specimen from Timor-Leste collected in 1999, identified as *Cx. tritaeniorhynchus*. Two specimens of *Culex* (*Culex*) *sitiens* Wiedemann, 1828 and one *Cx. crinicauda* were also included in the study, as these two species are the most likely to be confused with undescribed Australian Vishnui subgroup members.

CO_2_-baited encephalitis vector surveillance (EVS) traps were set in late afternoon and trapped adult mosquitoes were collected the following morning. The traps consist of an insulated bucket baited with 1 kg of dry ice, a suction fan powered by two ‘D’ cell batteries, a ‘grain of wheat’ light and a rigid collection container (volume: 4 l, diameter: 220 mm) fitted with a muslin sleeve and very fine wire mesh vents [17]. Adult specimens were dry mounted, vouchered and preliminarily identified using morphology. A single leg or larval segments from each specimen collected were transferred into vials containing 100% ethanol for DNA barcoding.

### DNA extraction and sequencing

Total DNA was extracted from mosquito legs or larval segments using the Qiagen DNEasy Blood and Tissue Kit (Qiagen Pty Ltd., Chadstone, VIC, Australia) and eluted into 50 μl TE buffer. DNA barcodes for the COI gene were obtained for each specimen using PCR primers LepF1 (5′-attcaaccaatcataaagatattgg-3′) and LepR1 (5′-taaacttctggatgtccaaaaaatca-3′), and for older samples in combination with internal barcode primers MF1 (5′-gctttcccacgaataaataata-3′) and MR1 (5′-cctgttccagctccattttc-3′) [18].

DNA was amplified in a total PCR reaction volume of 50 μl containing 400 nM of each primer, 200 μM dNTP, 2.5 mM MgCl_2_, 1 μl DNA extract (< 1 ng DNA), Q solution, 1× supplied buffer and 1 U *Taq* (*Taq* PCR Core Kit; Qiagen Pty Ltd.) using the following cycling conditions: denaturation at 94 °C, 2 min; then 94 °C/1 min, 45 °C/1 min, 72 °C/1 min for 5 cycles; followed by 94 °C/1 min, 50 °C/1 min, 72 °C/1 min for 35 cycles; with a final incubation step at 72 °C for 10 min. The presence of PCR products was confirmed by agarose gel electrophoresis before purification and elution into 32 μl EB buffer using the QIAquick PCR Purification Kit (Qiagen Pty Ltd.). PCR products were sent for Sanger sequencing using an ABI 3730xl system by Macrogen Inc. (Seoul, South Korea).

### Sequence divergence and phylogenetic analysis

The COI sequences for a total of 38 *Culex* (*Culex*) species were obtained (GenBank Accession Numbers MW809416–MW809453; Additional file 1: Table S1) and aligned in MEGA X [19]. Additional sequences were sourced from GenBank to cover: (i) a wide geographical range of *Cx. tritaeniorhynchus* (i.e. specimens collected from China, India, Japan, Pakistan, United Arab Emirates and Vietnam); (ii) related species from the Vishnui subgroup (*Cx. pseudovishnui* and *Cx. vishnui*); (iii) a range of *Culex* (*Culex*) species: *Culex* (*Culex*) *annulirostris* Skuse, 1889,* Culex* (*Culex*) *australicus* Dobrotworsky & Drummond, 1953, *Cx. crinicauda, Culex* (*Culex*) *gelidus* Theobald, 1901*, Culex* (*Culex*) *globocoxitus* Dobrotworsky, 1953, *Culex* (*Culex*) *molestus* Forsskål, 1775, *Culex* (*Culex*) *palpalis* Taylor, 1912,* Cx. sitiens*,* Culex* (*Culex*) *quinquefasciatus* Say, 1823, all occurring in Australia, and the exotic species *Culex* (*Culex*) *annulioris* Theobald, 1901, *Culex* (*Culex*) *mimeticus* Noè, 1899,* Culex* (*Culex*) *orientalis* Edwards, 1921,* Culex* (*Culex*) *pervigilans* Bergroth, 1889 and *Culex* (*Culex*) *vagans* Wiedemann, 1828); (iv) widespread species that also occur in Australia that have been reported in previous phylogenetic analyses as recovering within the *Culex* (*Culex*) clade: *Culex* (*Oculeomyia*) *bitaeniorhynchus* Giles, 1901 and *Lutzia* (*Metalutzia*) *halifaxi* (Theobald, 1903) [20, 21]; (v) and the chosen outgroup, *Culex* (*Neoculex*) *fergusoni* (Taylor, 1914).

Phylogenetic analysis was performed on the CSIRO Pearcey high-performance computing cluster using IQ-TREE version 2.0.6 [22], with 1000 ultrafast bootstrap replicates [23] and the best partitioning scheme [24] implementing a separate partition model for each codon position as follows: position 3, TN {40.5634,16.4236} + F {0.462515,0.0346759,0.0197159,0.483093} + G4 {0.863244}; position 1, TNe {4.35032,70.0487} + FQ + G4 {0.147506}, and position 2, F81. Nodes with ultrabootstrap support of < 50% were collapsed in the final tree using Interactive Tree of Life version 6.1 [25]. In cases where a species had multiple specimens sharing identical haplotypes, a representative specimen was chosen to include in the phylogenetic analysis, with the clade number annotated on the phylogeny (as specified in Additional file 1: Table S1). Percentage identity of the DNA barcodes was calculated using the blastn suite in BLAST (basic local alignment search tool) [26] in GenBank (https://blast.ncbi.nlm.nih.gov/Blast.cgi) for the Australian specimens, focussing on *Cx. tritaeniorhynchus* to provide insights into the potential origin of the vector.

### Morphological identification

After a leg was removed for DNA extraction, pinned adult specimens were examined under a Zeiss dissecting microscope (Carl Zeiss AG, Jena, Germany) and identified using the key to adult females of Australasian *Culex* (*Culex*) provided by Marks in Lee et al. [2]. Diagnostic morphological features were identified after comparing recently collected specimens to reference material held in the CSIRO Australian National Insect Collection, Canberra, and Elizabeth ‘Pat’ Marks mosquito collection held at the Queensland Museum, Brisbane. A taxonomic key was prepared to separate adult females of *Cx. tritaeniorhynchus* from morphologically similar species from the NT.

Photographs were taken on a BK Imaging—PLUS Lab System (Visionary Digital, Hollywood, CA, USA) using a Canon 65 mm lens (Canon Inc., Tokyo, Japan) stacked in Zerene Stacker v 1.0 software and processed in Adobe Photoshop CS6 (Adobe Inc., San Jose, CA, USA) to obtain a fully-sharpened image. Morphological terminology follows Harbach and Knight [27, 28].

## Results

### Molecular diagnostics

**Fig. 2:**
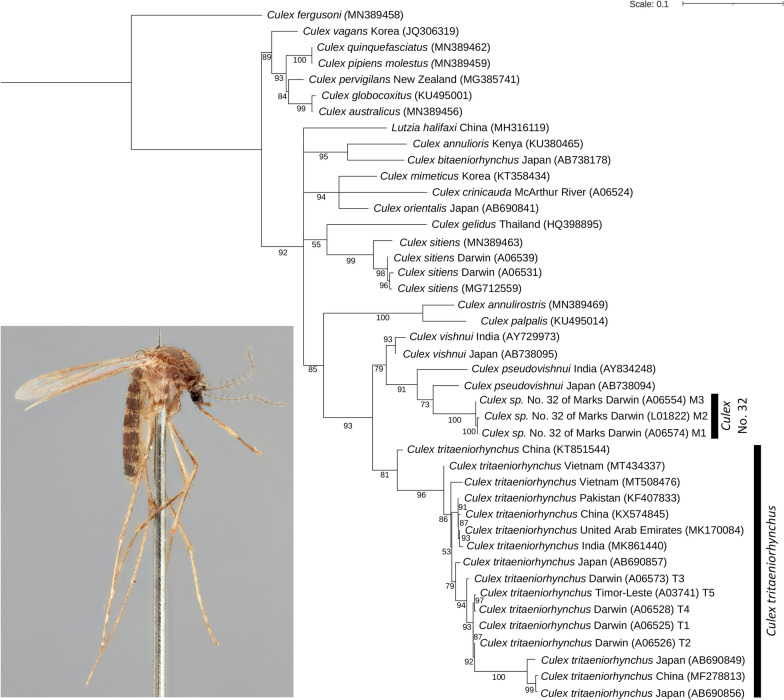
Cytochrome *c* oxidase subunit 1 (COI) phylogeny of *Culex* (*Culex*) species using maximum likelihood and a best-fit partitioning scheme in IQ-TREE. Ultrafast bootstrap values are shown at nodes. Specimens are collected from Australia unless labelled otherwise. Taxon information for samples sequenced in this study is presented in Additional file 1: Table S1

Standard length (658 bp) COI DNA barcodes were obtained for mosquitoes preliminarily identified using morphology as belonging to the Vishnui subgroup. These were collected between 1999 and 2020 from the NT (*n* = 34) and Timor-Leste (*n* = 1) (Additional file 1: Table S1). More than half (*n* = 19) of the Vishnui subgroup specimens sequenced from the NT were identified by COI barcoding as *Cx. tritaeniorhynchus*, sharing 98.2–98.8% nucleotide identity with records from Japan and/or Pakistan using BLAST in GenBank. Four different haplotypes were observed for the Australian *Cx. tritaeniorhynchus* specimens: haplotype T1 (*n* = 7: Katherine, Leanyer (Darwin urban), RAAF Base Darwin); haplotype T2 (*n* = 4: Howard Springs (Darwin region), Leanyer, RAAF Base Darwin); haplotype T3 (*n* = 7: Darwin International Airport, Howard Springs, Karama (Darwin urban), Leanyer, RAAF Base Darwin), and; haplotype T4 (*n* = 1: Howard Springs) (Additional file 1: Table S1; Fig. 2).

The *Cx. tritaeniorhynchus* specimens from the NT were most similar (99.7% nucleotide identity) to a specimen sequenced from Dili, Timor-Leste, collected in December 1999. One specimen identified as *Cx. crinicauda* was collected from the McArthur River Mine, located 900 km south-east of Darwin, and shared 95.1% nucleotide identity to a record of *Culex* (*Culex*) *orientalis* Edwards, 1921 from Japan, and 94.4% to a record of *Culex* (*Culex*) *mimeticus* Noè, 1899 also from Japan. The remaining 14 specimens collected from the Darwin region (subsequently confirmed morphologically as *Culex* sp. No. 32 of Marks), shared 96.0–96.4% nucleotide identity with records of *Cx. pseudovishnui* from Japan, and comprised three haplotypes: haplotype M1 (*n* = 12: Holtze [Darwin region], Karama, Leanyer, Lee Point [Darwin urban], Marrara [Darwin urban], Tiwi [Darwin urban], Winnellie [Darwin urban]); haplotype M2 (*n* = 1: Howard Springs); and haplotype M3 (*n* = 1: Leanyer) (Additional file 1: Table S1; Fig. 2).

Tree-based identification using maximum likelihood estimation revealed that *Cx. tritaeniorhynchus* recovered as a moderately supported monophyletic clade (ultrafast bootstrap support = 81%; Fig. 2). Sequence variation did not give any indication of geographic structure, as multiple specimens from each country recovered as paraphyletic. For instance, specimens from Australia were not monophyletic, but instead grouped within the larger *Cx. tritaeniorhynchus* clade of Darwin + ([Darwin + Timor] + Darwin + [Darwin + {Japan + (China + Japan)}]). The Vishnui subgroup recovered as a strongly supported monophyletic clade (ultrabootstrap support = 93%), grouping as *Cx. tritaeniorhynchus* + (*Cx. vishnui* + [*Cx. pseudovishnui* + *Culex* sp. No. 32 of Marks]) (Fig. 2). However, *Cx. crinicauda* was excluded from the Vishnui subgroup, which instead recovered as a strongly supported polytomic clade (ultrabootstrap support = 94%) with *Cx. orientalis* and *Cx. mimeticus*. The undescribed species *Culex* sp. No. 32 of Marks formed a strongly supported monophyletic group (ultrabootstrap support = 100%), sister to *Cx. pseudovishnui* (Fig. 2).

### Morphological identification

**Fig. 3:**
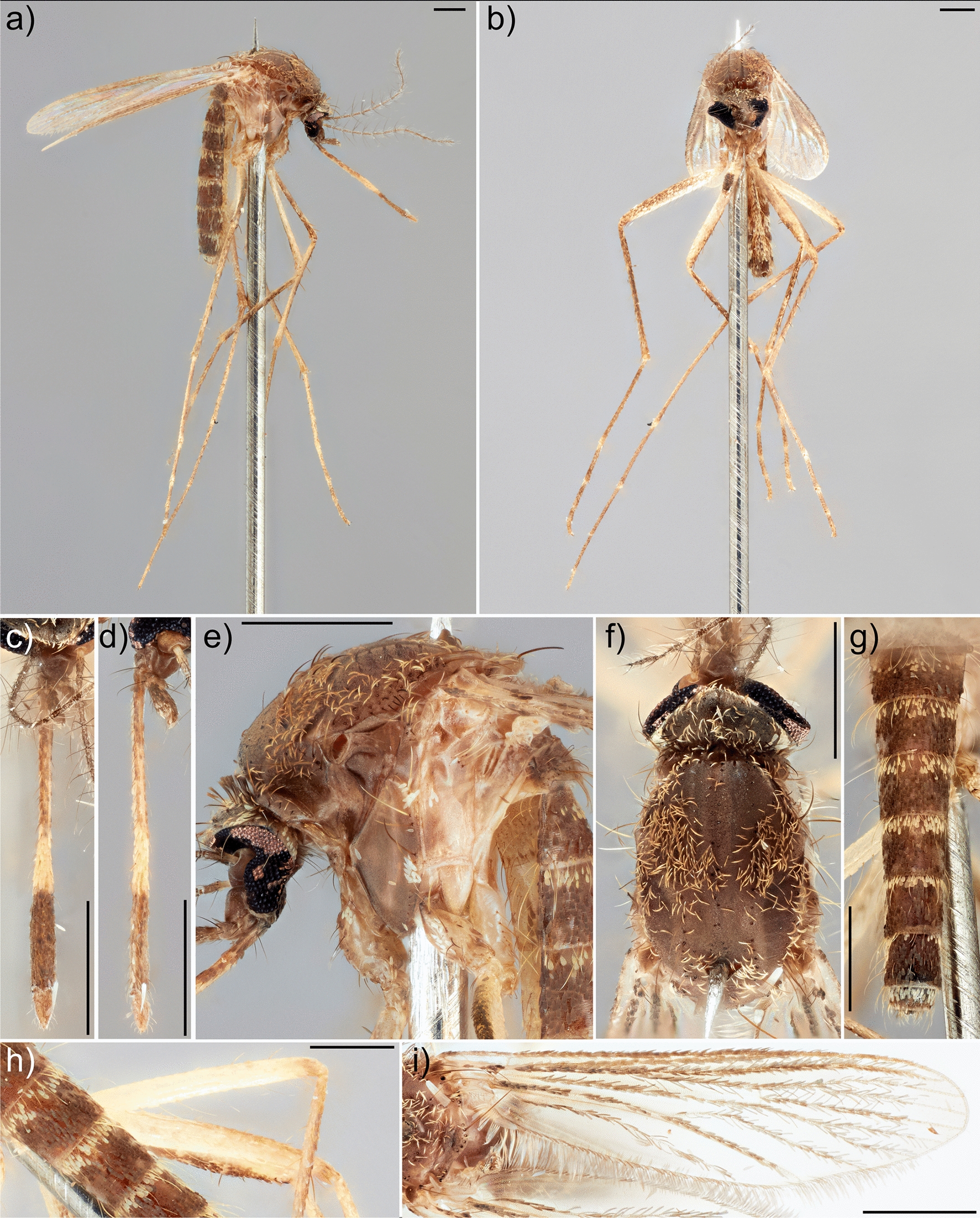
*Culex* (*Culex*) *tritaeniorhynchus*, female (NT Health A06525). **a** Body, lateral; **b** body, frontal; **c** proboscis, dorsal; **d** proboscis, lateral; **e** thorax, lateral; **f** scutum; **g** abdomen, dorsal; **h** posterior of legs, hind (top) and mid (bottom); **i** wing, dorsal. Scale bars: 0.5 mm. Collection label data: “NM08542 A06525/12°29′15″S 131°1′45″E/AUS., NT, Litchfield Shire/Stow Road, behind Manigur/Coll: 27-Feb-2020/Coll: A Roberts/Coll type: CO2”

**Fig. 4:**
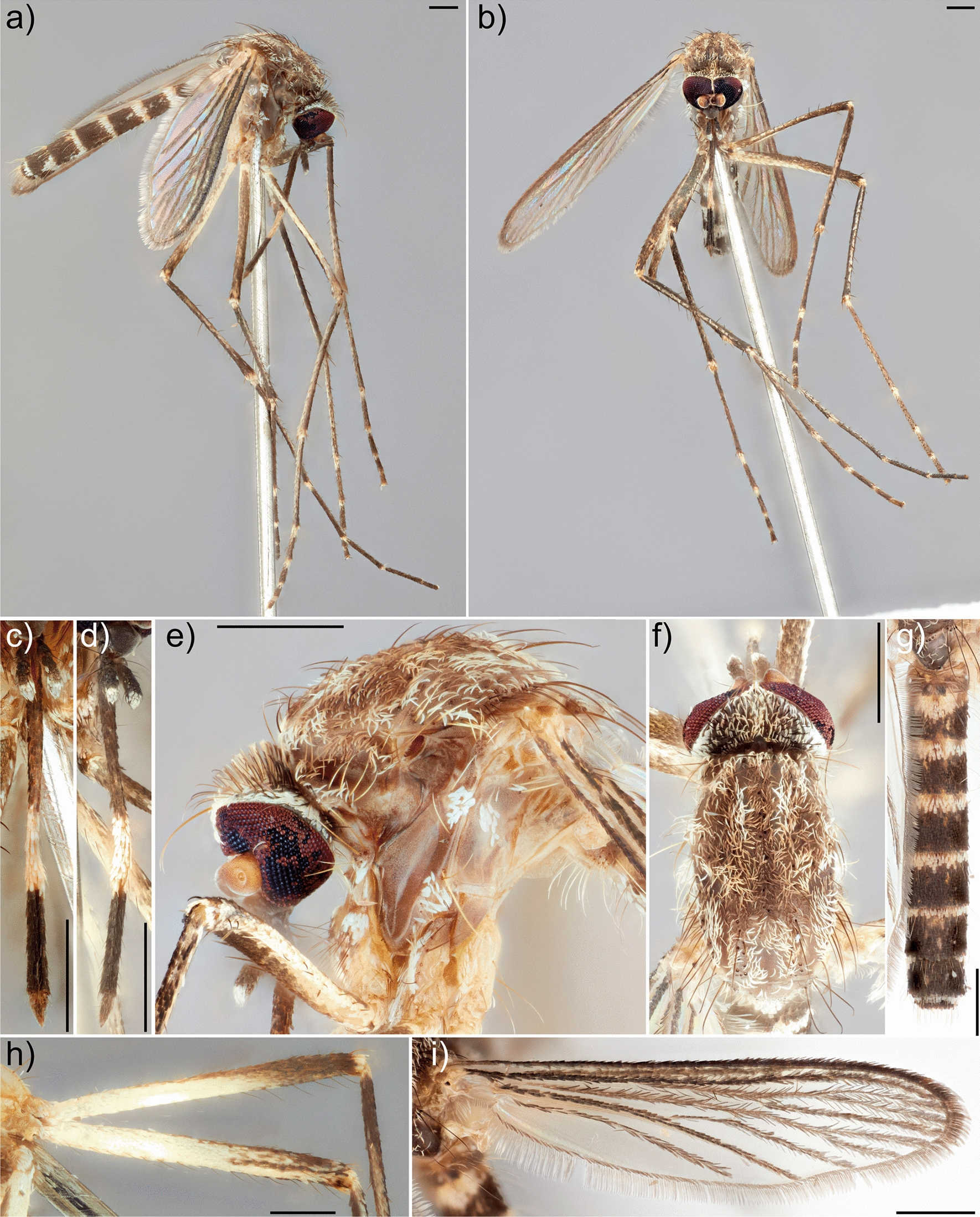
*Culex* (*Culex*) sp. No. 32 of Marks, female (NT Health A06575). **a** Body, lateral; **b** body, frontal; **c** proboscis, dorsal; **d** proboscis, lateral; **e** thorax, lateral; **f** scutum; **g** abdomen, dorsal; **h** posterior of legs, hind (top) and mid (bottom); **i** wing, dorsal. Scale bars: 0.5 mm. Collection label data: “NM08559 A06575/12°24′26″S 130°54′44″E/AUS., NT, Darwin/DM08 Marrara Round Swa/Coll: 14-May-2020/Coll: T Okazaki/Coll type: CO2”

Once species identify was confirmed using the COI gene, reference specimens were re-examined for informative morphological characters that could be used to diagnose species. Regarding the Australian *Culex* (*Culex*) fauna, *Cx. tritaeniorhynchus* is likely to be confused with the undescribed species *Culex* No. 32 of Marks, sharing the overall brown coloration, as well as narrow, pale banded scaling on the proboscis, abdomen and, to a lesser extent, the legs. The two species, however, can be separated based on the morphological characters provided in the following key to adult females of the Vishnui subgroup and morphologically similar *Cx. crinicauda* from the NT:

1. Occiput with erect forked scales predominantly white, sharply contrasting with cuticle; scutum with prominent, dense whitish scaling, sharply contrasting with cuticle... *crinicauda* Edwards, 1921

- Occiput with erect forked scales predominantly brown, more uniform with cuticle; scutum with predominantly pale brown scaling, if white scaling is present, it is usually dispersed, never forming dense patches... 2

2. Proboscis with very narrow pale band, approximately 0.1 times length of proboscis, not sharply contrasting remaining proboscis; pale-brown species... sp. No. 92 of Marks (1982)

- Proboscis with well-defined pale band, at least 0.2 times length of proboscis, sharply contrasting remaining proboscis; darker species... 3

3. Legs pale brown, posterior mid and hind femora almost entirely pale yellowish to white, anterior surface of mid tibiae almost uniform brown, without pale line (Fig. 3b, h); vertex with erect forked scales predominantly dark (Fig. 3e); scutum with uniform dull brown scaling, without obvious patches of whitish scales (Fig. 3e, f); pleura with predominantly bare scaling on proepisternum, occasionally with only a few whitish scales, reduced on upper and lower mesokatepisternum, upper mesanepimeron, and anterior surface of forecoxa (Fig. 3e)... *tritaeniorhynchus* Giles, 1901

- Legs dark brown with strongly contrasting pale banding, posterior mid and hind femora dark brown on apical third, strongly contrasting pale yellowish white basal two thirds, anterior surface of mid tibiae with a longitudinal pale scale patch strongly contrasting dark scales (Fig. 4b, h); vertex with erect forked scales pale medially, becoming dark laterally (Fig. 4e); scutum scaling pale brown, with contrasting whitish scaling present at dorsocentral areas, scutal angle, prescutellar and supra-alar areas (Fig. 4e, f); pleura with relatively dense scaling, broad whitish on proepisternum, upper and lower mesokatepisternum, upper mesanepimeron, and anterior surface of forecoxa (Fig. 4e)... sp. No. 32 of Marks (1982).

## Discussion

*Culex tritaeniorhynchus* appears to be established in the NT, with confirmed collection records from the Darwin region, extending 270 km further SE to Katherine (Fig. 1). Tree-based identification using a 658-bp COI barcoding region demonstrated moderate support for the monophyly of *Cx. tritaeniorhynchus* collected throughout the world, including Australia, China, India, Japan, Pakistan, Timor-Leste, United Arab Emirates and Vietnam (Fig. 2). Sequence divergence results indicate that the Australian *Cx. tritaeniorhynchus* population most likely originated from Timor-Leste, sharing 99.7% nucleotide similarity. Geographically, Timor-Leste is separated by approximately 620 km from Australia by the Timor Sea and is the closest known population of *Cx. tritaeniorhynchus* to the Darwin region.

While the introduction pathways are unconfirmed, it is plausible that *Cx. tritaeniorhynchus* may have travelled to Australia from Timor-Leste* via* windblown adult mosquitoes, given the relatively short distance of 465 km between Timor-Leste and Melville Island near Darwin, and that *Cx. tritaeniorhynchus* has been previously recorded as flying 200–500 km over sea waters in the Northwest Pacific [29]. Alternatively, the vector may have arrived in Australia with adults being transported on board aircraft, or most likely as larvae and/or pupae inadvertently stowed on cargo ships. In one study documenting the number of mosquitoes detected on ships arriving in China from abroad, *Cx. tritaeniorhynchus* was one of the most common mosquito species recorded [10, 30]. Moreover, transportation* via* shipping vessels has been identified as the main point of entry for the introductions and subsequent establishment of other *Culex* (*Culex*) species into Australia, including *Cx. molestus* (by US forces during the Second World War) and *Cx. quinquefasciatus* (by European colonists, US whalers or international trade) [10, 31].

The Australian members of subgenus *Culex* remain difficult to identify using morphology alone, with accurate species identification hindered by the presence of undescribed [11] or potential cryptic species [13]. We demonstrate that DNA barcoding is useful for identifying members of the Vishnui subgroup from the NT and present the first genetic sequences to be provided for *Cx. crinicauda* and *Culex* sp. No. 32 of Marks. The Vishnui subgroup recovered as a strongly supported monophyletic group in the COI phylogeny (Fig. 2), excluding *Cx. crinicauda* which instead formed a clade with *Cx. orientalis* and *Cx. mimeticus*. Although *Cx. crinicauda* was previously proposed to be part of the subgroup [11], our results support the decision of Lee et al. [2] to exclude it from the Vishnui subgroup. The monophyly of the Vishnui subgroup is also supported by the COI phylogeny presented by Karthika et al. [9]. The undescribed species *Culex* sp. No. 32 of Marks also formed a strongly supported monophyletic group in our phylogenetic analysis, sister to *Cx. pseudovishnui*, demonstrating that it is in fact a valid species in need of formal taxonomic description. A modern taxonomic revision combining morphology and molecular data is warranted for the Australian mosquitoes to improve species identification and the detection of future incursions of invasive species.

*Culex tritaeniorhynchus* occurs in sympatry with other species from the Vishnui subgroup from the NT. It is most likely to be confused morphologically with the undescribed species *Culex* sp. No. 32 of Marks (Fig. 4). Nevertheless, both species can be reliably identified using the COI gene, as each species formed distinct monophyletic clades in the molecular phylogeny (Fig. 2). Adult females of *Cx. tritaeniorhynchus* can be distinguished from all other species of the Vishnui subgroup from the NT by a combination of the following traits: vertex with erect forked scales mostly dark; scutum with uniform dull brown scaling; legs with posterior surface of mid and hind femora almost entirely pale yellowish to white, anterior surface of mid tibiae almost uniform brown and without a longitudinal pale scale patch; and pleura with proepisternum without scaling, and reduced scaling on upper and lower mesokatepisternum, upper mesanepimeron and anterior surface of forecoxa (Fig. 3).

The vector may have been first introduced into Australia several decades ago, since *Cx. tritaeniorhynchus* larvae were reportedly collected during larval surveys from Darwin and the Kimberley Research Station in the state of Western Australia in the 1950s [32, 33]. However, the larvae were not illustrated and the whereabouts of the original specimens are unknown. Moreover, larvae of *Cx. tritaeniorhynchus* and *Cx.* sp. No. 32 of Marks are very similar morphologically, sharing similar pecten spines, comb scaling and branching setae of the head [34, 35]. Therefore, it is possible that these early larval records of *Cx. tritaeniorhynchus* were misidentifications of *Cx.* sp. No. 32 of Marks that was unknown at the time.

*Culex tritaeniorhynchus* is the most recent exotic *Culex* (*Culex*) species to be detected in Australia in more than 20 years. *Culex gelidus* (distributed in India and Southeast Asia), also a known JEV vector, was first detected in Australia in 1999 and was introduced* via* aircraft in northern Queensland, before spreading further and becoming established in the NT and northern Western Australia [36–38]. Six JEV vectors from the *Culex* (*Culex*) are now known to occur in Australia:* Cx. annulirostris*,* Cx. gelidus*,* Cx. quinquefasciatus*,* Cx. sitiens*,* Cx. tritaeniorhynchus* and *Culex* (*Culex*) *whitmorei* (Giles, 1904) [39]*.*

Eradication programmes of mosquito vectors are cost prohibitive and further complicated by widespread species [40, 41]. However, following the detection of *Cx. tritaeniorhynchus* in the Darwin and Katherine regions (Fig. 1), it appears that the species is already widely established, therefore elimination is most likely unfeasible. The full distribution of Australian breeding sites of *Cx. tritaeniorhynchus* is unknown, although breeding habitats appear to be broad overseas, including temporary and semi-permanent shaded ground pools [1, 10], which are common in the NT. Moreover, vertical transmission of JEV has been noted in F_1_ progeny of *Cx. tritaeniorhynchus*, as has the ability of females to overwinter and estivate in colder months [10, 42, 43], both of which enhance the threat of the JEV vector becoming established and expanding into suitable environments [1]. Increased larval and adult surveys in the NT and northern Western Australia are needed to confirm the current geographical spread and continued presence of the species. Historically collected specimens held in mosquito surveillance and entomological collections may be useful in tracing the origins and first detection of the species in Australia, especially given the recent advances of sequencing DNA from museum mosquito specimens [44].

An estimated 2.3–6.3 million feral pigs occur in Australia [45], with 6.1 pigs km^−2^ estimated from the Mary River region in the NT alone [46]. As pigs are known amplifier hosts for JEV [37], the establishment of *Cx. tritaeniorhynchus* in Australia may be considered a public health concern due to the abundance of feral pigs occurring across northern Australia, which may increase infection rates and potentially lead to emerging JEV outbreaks [3, 37].

Recent vector competence testing has shown that possums and the black flying fox *Pteropus alecto* are potential amplifying hosts for JEV in Australia, compared to those considered to be poor hosts, such as the eastern grey kangaroos, agile wallabies and tammar wallabies [22, 47–49]. Van den Hurk et al. [3] suggested that flying foxes could play a prominent role in the transmission of JEV into northern Australia, since thousands of individuals migrate to Australia from Torres Strait and New Guinea where the virus is more prevalent. Future vector competency testing should be conducted to assess whether JEV could be spread by other Australian members of the Vishnui subgroup, including *Culex* sp. No. 32 of Marks.

Although JEV is relatively rare and yet to be established in Australia [3, 12], northern Australia is a JEV risk area [39]. The first outbreak of the arbovirus was last detected in 1995 in humans and pigs from the Torres Strait and Cape York peninsula, northern Queensland, most likely introduced by migratory birds or windblown mosquitos from New Guinea and amplified by the native JEV vector mosquito *Cx. annulirostris* [6, 50]. Therefore, the additional JEV vector *Cx. tritaeniorhynchus* must now be continuously monitored for to prevent the potential health risk of transmitting this exotic disease in Australia.

## Conclusions

The JEV vector *Cx. tritaeniorhynchus* is detected for the first time in Australia from the Darwin and Katherine regions in the NT. Molecular analysis of the COI gene confirmed the identify of specimens collected from the NT as belonging to *Cx. tritaeniorhynchus*, most likely originating from Timor-Leste. Accurate species identification of exotic species with known affinities for disease transmission is essential for improving the monitoring of high-risk mosquito species to better predict and manage emerging mosquito-borne diseases in Australia.

## Supplementary Information

**Additional file 1: Table S1**. Australian *Culex *(*Culex*) spp. mosquitoes sequenced in this study, focusing on the Vishnui subgroup.

## Abbreviations

BLAST: Basic local alignment search tool
COI: Cytochrome *c* oxidase subunit 1
CSIRO: Commonwealth Scientific and Industrial Research Organisation
JEV: Japanese Encephalitis virus
ME: Medical Entomology Top End Health Service NT mosquito surveillance programme
NT: Northern Territory

## Declarations

### Availability of data and materials

Sequence data that support the findings of this study have been deposited in GenBank with the accession codes provided in Additional file 1: Table S1.
