# Supplementary material for: Detection of the Japanese encephalitis vector mosquito Culex tritaeniorhynchus in Australia using molecular diagnostics and morphology
Source: Parasit Vectors. 2021 Aug 18;14:411. doi: 10.1186/s13071-021-04911-2 (PMC8371801; doi:10.1186/s13071-021-04911-2)
Supplement: Supplementary file 1 — Additional file 1: Table S1. Australian Culex (Culex) spp. mosquitoes sequenced in this study, focusing on the Vishnui subgroup. [file 13071_2021_4911_MOESM1_ESM.docx]

**Additional file 1: Table S1.**Table S1 Australian *Culex* (*Culex*) spp. mosquitoes sequenced in this study, focusing on the Vishnui subgroup ^a^

| Voucher | Tissue | Collection date | Location | COI clade | Species identification | GenBank accession No. |
| --- | --- | --- | --- | --- | --- | --- |
| A06524 | Leg | 12-Feb-20 | McArthur River, NT | **-** | *Culex crinicauda* | MW809440 |
| A06531 | Leg | 10-Feb-20 | RAAF Base, Darwin, NT | - | *Culex sitiens* | MW809418 |
| A06539 | Leg | 10-Feb-20 | Airport, Darwin, NT | - | *Culex sitiens* | MW809436 |
| A06574 | Leg | 27-Mar-19 | Holtze, Darwin, NT | M1 | *Culex* sp. No. 32 of Marks | MW809448 |
| A06551 | Leg | 31-Mar-20 | Karama, Darwin, NT | M1 | *Culex* sp. No. 32 of Marks | MW809429 |
| A06553 | Leg | 25-Mar-20 | Leanyer, Darwin, NT | M1 | *Culex* sp. No. 32 of Marks | MW809427 |
| A06555 | Leg | 3-Mar-20 | Lee Point, Darwin, NT | M1 | *Culex* sp. No. 32 of Marks | MW809442 |
| A06556 | Leg | 31-Mar-20 | Lee Point, Darwin, NT | M1 | *Culex* sp. No. 32 of Marks | MW809443 |
| A06557 | Leg | 31-Mar-20 | Lee Point, Darwin, NT | M1 | *Culex* sp. No. 32 of Marks | MW809444 |
| A06558 | Leg | 31-Mar-20 | Lee Point, Darwin, NT | M1 | *Culex* sp. No. 32 of Marks | MW809445 |
| A06575 | Leg | 14-May-20 | Marrara, Darwin, NT | M1 | *Culex* sp. No. 32 of Marks | MW809449 |
| A06576 | Leg | 14-May-20 | Marrara, Darwin, NT | M1 | *Culex* sp. No. 32 of Marks | MW809450 |
| A06577 | Leg | 14-May-20 | Marrara, Darwin, NT | M1 | *Culex* sp. No. 32 of Marks | MW809451 |
| A06548 | Leg | 11-Mar-20 | Tiwi, Darwin, NT | M1 | *Culex* sp. No. 32 of Marks | MW809441 |
| A06572 | Leg | 31-Mar-20 | Tiwi, Darwin, NT | M1 | *Culex* sp. No. 32 of Marks | MW809446 |
| A06552 | Leg | 25-Mar-20 | Winnellie, Darwin, NT | M1 | *Culex* sp. No. 32 of Marks | MW809426 |
| L01822 | Larval segment | 7-May-20 | Howard Springs, Darwin, NT | M2 | *Culex* sp. No. 32 of Marks | MW809453 |
| A06554 | Leg | 25-Mar-20 | Leanyer, Darwin, NT | M3 | *Culex* sp. No. 32 of Marks | MW809428 |
| A06525 | Leg | 27-Feb-20 | Howard Springs, Darwin, NT | T1 | *Culex tritaeniorhynchus* | MW809432 |
| A06541 | Leg | 14-Feb-20 | Katherine, NT | T1 | *Culex tritaeniorhynchus* | MW809430 |
| A06521 | Leg | 18-Feb-20 | Leanyer, Darwin, NT | T1 | *Culex tritaeniorhynchus* | MW809437 |
| A06529 | Leg | 10-Feb-20 | RAAF Base, Darwin, NT | T1 | *Culex tritaeniorhynchus* | MW809416 |
| A06530 | Leg | 10-Feb-20 | RAAF Base, Darwin, NT | T1 | *Culex tritaeniorhynchus* | MW809417 |
| A06532 | Leg | 10-Feb-20 | RAAF Base, Darwin, NT | T1 | *Culex tritaeniorhynchus* | MW809419 |
| A06534 | Leg | 10-Feb-20 | RAAF Base, Darwin, NT | T1 | *Culex tritaeniorhynchus* | MW809421 |
| A06526 | Leg | 27-Feb-20 | Howard Springs, Darwin, NT | T2 | *Culex tritaeniorhynchus* | MW809433 |
| A06523 | Leg | 18-Feb-20 | Leanyer, Darwin, NT | T2 | *Culex tritaeniorhynchus* | MW809439 |
| A06533 | Leg | 10-Feb-20 | RAAF Base, Darwin, NT | T2 | *Culex tritaeniorhynchus* | MW809420 |
| A06537 | Leg | 10-Feb-20 | RAAF Base, Darwin, NT | T2 | *Culex tritaeniorhynchus* | MW809424 |
| A06573 | Leg | 14-Apr-20 | Airport, Darwin, NT | T3 | *Culex tritaeniorhynchus* | MW809447 |
| A06527 | Leg | 27-Feb-20 | Howard Springs, Darwin, NT | T3 | *Culex tritaeniorhynchus* | MW809434 |
| A06540 | Leg | 3-Mar-20 | Karama, Darwin, NT | T3 | *Culex tritaeniorhynchus* | MW809431 |
| A06522 | Leg | 18-Feb-20 | Leanyer, Darwin, NT | T3 | *Culex tritaeniorhynchus* | MW809438 |
| A06535 | Leg | 10-Feb-20 | RAAF Base, Darwin, NT | T3 | *Culex tritaeniorhynchus* | MW809422 |
| A06536 | Leg | 10-Feb-20 | RAAF Base, Darwin, NT | T3 | *Culex tritaeniorhynchus* | MW809423 |
| A06538 | Leg | 10-Feb-20 | RAAF Base, Darwin, NT | T3 | *Culex tritaeniorhynchus* | MW809425 |
| A06528 | Leg | 27-Feb-20 | Howard Springs, Darwin, NT | T4 | *Culex tritaeniorhynchus* | MW809435 |
| A03741 | Leg | 16-Dec-99 | Dili, Timor-Leste | T5 | *Culex tritaeniorhynchus* | MW809452 |

^a^ GenBank numbers and country of origin for additional specimens and outgroups used in the phylogenetic analysis are presented in Fig. 2.
